# The Hospital Steward’s Manual; &c.

**Published:** 1862-10

**Authors:** 


THE HOSPITAL STEWARD'S MANUAL; FOR THE INSTRUCTION OF HOSPITAL STEWARDS, WARD-MASTERS, AND ATTENDANTS, IN THEIR SEVERAL DUTIES. Prepared in strict accordance with existing regulations and the customs of service in the Armies of the United States of America; and rendered authoritative by order of the Surgeon-General. By JOSEPH JANVIER WOODWARD, M.D., Assistant-Surgeon U.S.A., Member of the Academy of Natural Sciences of Philadelphia, &c. Philadelphia: J. B. LIPPINCOTT & Co. 1862.

This is an excellent little manual of 324 small-sized octavo pages, containing just what its title indicates. It is printed on good paper, in fair type, and should be in the hands of all the Hospital Stewards, Nurses, and many of the Surgeons of the Army.

For sale at the Bookstore of S. C. GRIGGS & Co., 41 Lake Street, Chicago.

